# Electrochemical Deposition of Nanomaterials for Electrochemical Sensing

**DOI:** 10.3390/s19051186

**Published:** 2019-03-08

**Authors:** Domenica Tonelli, Erika Scavetta, Isacco Gualandi

**Affiliations:** Department of Industrial Chemistry “Toso Montanari”, Viale del Risorgimento 4, 40136 Bologna, Italy; erika.scavetta2@unibo.it (E.S.); isacco.gualandi2@unibo.it (I.G.)

**Keywords:** electrochemical deposition, electrochemical synthesis, electrochemical sensors, gold nanomaterials, layered double hydroxides, nanostructured polymers, molecularly imprinted polymers

## Abstract

The most commonly used methods to electrodeposit nanomaterials on conductive supports or to obtain electrosynthesis nanomaterials are described. Au, layered double hydroxides (LDHs), metal oxides, and polymers are the classes of compounds taken into account. The electrochemical approach for the synthesis allows one to obtain nanostructures with well-defined morphologies, even without the use of a template, and of variable sizes simply by controlling the experimental synthesis conditions. In fact, parameters such as current density, applied potential (constant, pulsed or ramp) and duration of the synthesis play a key role in determining the shape and size of the resulting nanostructures. This review aims to describe the most recent applications in the field of electrochemical sensors of the considered nanomaterials and special attention is devoted to the analytical figures of merit of the devices.

## 1. Introduction

The realization of modified electrodes has been of pivotal importance for the development of a new generation of electroanalysis devices with enhanced sensitivity and selectivity since the modifiers confer interesting properties to the support which can lead to a specific recognition and/or a pre-concentration of the analytes. Fundamental studies of such modified electrodes have been performed to obtain a better comprehension of the nature of charge transfer and charge transport processes inside thin films [1].

The methods to modify a conductive surface can involve adsorption, covalent bond formation, coating with previously synthesized materials, e.g., soluble polymers, or electrodeposition, when the modifiers can be electrosynthesized.

In recent years, nanomaterials have attracted much attention as suitable materials to modify the surface of the electrodes due to their intriguing physicochemical properties, which differ significantly from those displayed by the same bulk materials.

For example, nanomaterials possess exceptional electrical and catalytic properties, large surface-to-volume ratio (aspect ratio) and large number of adsorption-active sites which make them particularly suitable for analytical purposes [2]. The properties of nanoparticles strongly depend on their size and shape, so a synthetic procedure, controlling the growth and the morphology of the nanoparticles, is critical and appealing [3].

**Figure 1:**
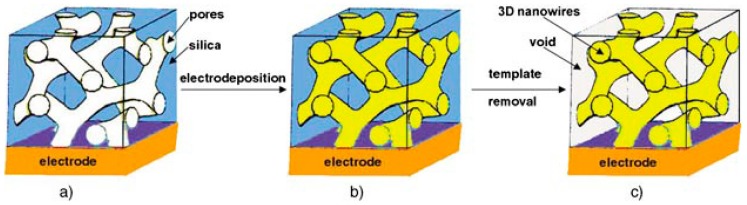
Schematic showing the formation of 3D continuous macroscopic metal or semiconductor nanowire networks by a templated electrodeposition technique. (**A**) 3D cubic mesoporous template, (**B**) 3D nanowire/silica nanocomposites, (**C**) 3D nanowire network. Images reproduced from Ref. [8] with permission.

Electrochemical deposition is an efficient procedure to prepare metal nanoparticles but it is usually less utilized than wet-chemical methods. This approach can sometimes display some limitations as to the nanomaterial dimensions and the allowed morphologies, but it shows a lot of advantages, particularly related to the rapid synthesis time, the absence of chemical reductants or oxidants, and of undesired by-products [4]. Furthermore, when the modifier film is directly deposited on the electrode it permits a better adhesion to be obtained [5]. Electrodeposition is widely applied using different electrochemical techniques, such as cyclic voltammetry, potential step and double-pulse deposition [6]. The possibility of a precise particle size control is achieved by adjusting current density or applied potential and electrolysis time [7]. Furthermore, combined with a template, electrochemical synthesis gives the opportunity to produce a variety of 3D networks, e.g., through mesoporous silica films (this is the case of noble metal nanowires, see Figure 1) [8].

With the introduction of colloidal silica, more complex nanostructures, such as sponge-like and grass-like morphologies, can be synthesized by varying the dimension and shape of the silica additives [9].

This review aims to describe the most recent applications as electrochemical sensors of supports modified with electrodeposited nanomaterials or obtained by electrosynthesis, belonging to two categories: inorganics and organics. In the former case metals, layered double hydroxides (LDHs) and metal oxides (and hybrids) have been taken into account, in the latter conducting, insulating and molecularly imprinted polymers (MIPs) have been considered.

## 2. Metal Nanoparticles

As far as metal nanomaterials are concerned, gold nanoparticles (Au-NPs) will be mainly taken into account since in the last decades they have been used for the fabrication of a lot of sensors due to their optimal conductivity, biological compatibility and high aspect ratio.

### 2.1. Au-NPs Electrosynthesis

From an electroanalytical point of view, Au-NPs show interesting size- and shape-dependent physicochemical properties. In the macroscopic phase, gold is considered an inert material; on the contrary, at the nanometer scale its chemical reactivity increases greatly, as a function of the size, shape, composition, morphology dispersion, and crystalline status [4].

Many chemical methods have been proposed for the preparation of Au-NPs with controlled size and shape [10], whereas electrochemical techniques are usually less employed [11]. As stated before, chemical synthesis generally provides NPs of almost any shape and size, while the electrochemical approach has some limitations as far as the size range and the morphologies that can be realized are concerned. The main advantages of electrochemical depositions are that the NPs are obtained already anchored to a surface easily and rapidly, with an inexpensive procedure and without chemical or binding agents [12], so they are more environmentally friendly than the one produced by chemical methods. Moreover, the nanomaterials do not need to be stabilized as required if the same materials are synthesized in solution. Therefore, ever increasing interest has been devoted to the development of effective electrochemical methods for the deposition of metal NPs [13].

Many studies have focused on the initial stages of the deposition in order to investigate in depth the nucleation and crystal growth mechanisms of the metal phase on the conductive support, usually a glassy carbon electrode (GCE). The steps of the Au-NPs deposition are first the electrochemical reduction of AuCl_4_^-^ salt, then the formation of ad-atoms, and, finally, the further growth of nanocrystals on the GCE. The overall surface area of gold, as well as the size and density of the nanocrystals, and surface texture can be modulated by changing the deposition conditions such as the salt concentration and the applied potential or the time of electroreduction [14].

The electrosynthesis of Au-NPs has been also carried out in the presence of additives, with the aim to control not only the size, but also the preferential crystallographic orientations of the gold nanoparticles. Au-NPs were electrodeposited on GC electrodes in the presence of two additives, i.e., cysteine and iodide ions at 100 μM concentration. The results were that in the former case the NPs were enriched in the Au(100) and Au(110) facets and displayed a relatively large dimension (300 nm), in the latter they were enriched in the Au(111) facets and possessed a relatively narrow size distribution range (10–40 nm) [15].

**Figure 2:**
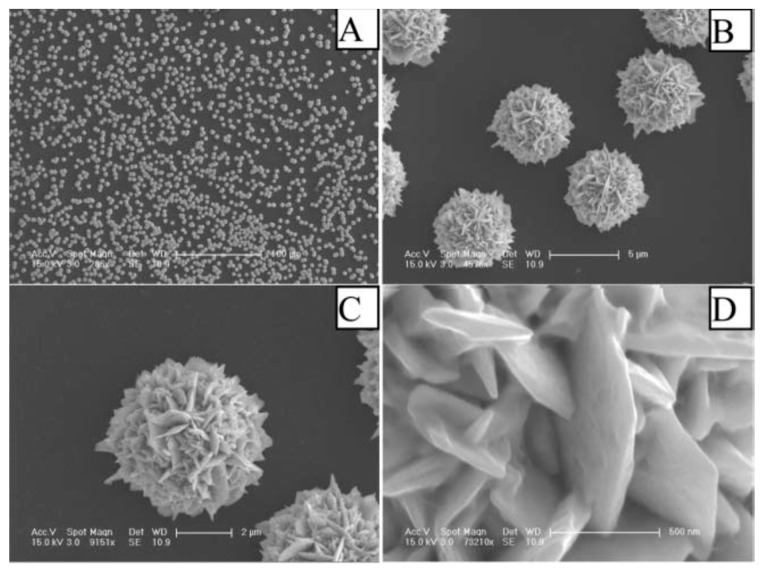
Typical FE-SEM images of the hierarchical flowerlike Au microstructures deposited on ITO substrate at different magnifications; The scale bars for (**A**–**D**) are 100, 5, 2 μm and 500 nm, respectively. Images reproduced from [16] by permission of The Royal Society of Chemistry.

Hierarchical flowerlike Au microstructures have been synthesized on indium tin-oxide (ITO) substrates without introducing any template or surfactant. These Au microstructures were composed of gold nanoplates or nanoprisms as building blocks and their diameter was dependent on the deposition time or the deposition potential. The electrodeposition process was carried out in 24.3 mM HAuCl_4_ solution at 0.5 V vs. Ag/AgCl for 30 min. Figure 2 shows SEM images of the as-prepared flowerlike structures at different magnifications [16].

Au-NPs resultiing from electrochemical reduction of AuCl_4_^−^ and packed within metalloporphyrin layers were fabricated in situ to obtain 3D multilayer films which showed good electrocatalytic activity for the two-electron reduction of oxygen. This property could be exploited to develop promising O_2_ sensors. Furthermore, it was also observed that the electrocatalytic efficiency was dependent on the number of layers, in particular it increased with the layers number [17].

Ionic liquids (ILs), particularly air and water stable ones, are very appealing due to their interesting physical properties. ILs allow for electrodeposition of many materials, e.g., Al and Mg, that cannot be obtained with classical electrosynthetic methods. Moreover, they can control the layer morphology through the effects of their large cations. A careful choice of IL cations can avoid the use of traditional templates [9]. As an example, nanocrystalline Cu and Al could be electrodeposited in 1-butyl-1-methylpyrrolidinium trifluoromethanesulfonate and 1-butyl-1-methylpyrrolidinium bis(trifluoromethylsulfonyl)amide, respectively, on classical solid supports without using additives [18]. Moreover, shape specificity can be controlled by changing the IL anions, the applied potential, water concentration, temperature, and the ratio of the precursor ion to the IL molecules [19].

Taking into account the crystallographic characteristics, high-index planes are particularly attractive for their higher density of active sites but are difficult to produce due to their lower stability. Depending on the ppm content of water in the IL bath, high-index faceted particles with star, nanothorn, and snowflake shapes could be obtained in the Au NPs synthesis. All the shapes showed an improved activity for the electrocatalytic reduction of hydrogen peroxide if compared to bulk Au, but the star-shaped NPs displayed a 14-fold higher activity [20].

### 2.2. Au Nanostructures Electrosynthesis

In order to produce nanoporous metal films, methods based on alloy corrosion have been proposed. Dealloying of metal alloys such as Ag–Au can produce nanoporous metals with interesting characteristics. By optimizing the Au content of the alloy, applying a potential below the threshold value and increasing the temperature of the electrolyte, it was possible to prepare dealloyed membranes displaying very good mechanical stability [21]. In the case of Au, a nanoporous film (NPGF) on a gold electrode was produced by applying multicyclic electrochemical alloying/dealloying in an electrolyte containing ZnCl_2_ and benzyl alcohol. In the cathodic potential scan, Zn was first electrodeposited on the Au support, and later an Au−Zn alloy was formed at high temperature. In the following anodic potential scan, dealloying of Zn occurred, resulting in a nanostructured Au film with nanopores. The film displayed a much higher surface area, a very high roughness factor, and better electron transport than the ones exhibited by the bulk Au. It was applied for the selective quantification of dopamine in the presence of ascorbic acid [22].

**Figure 3:**
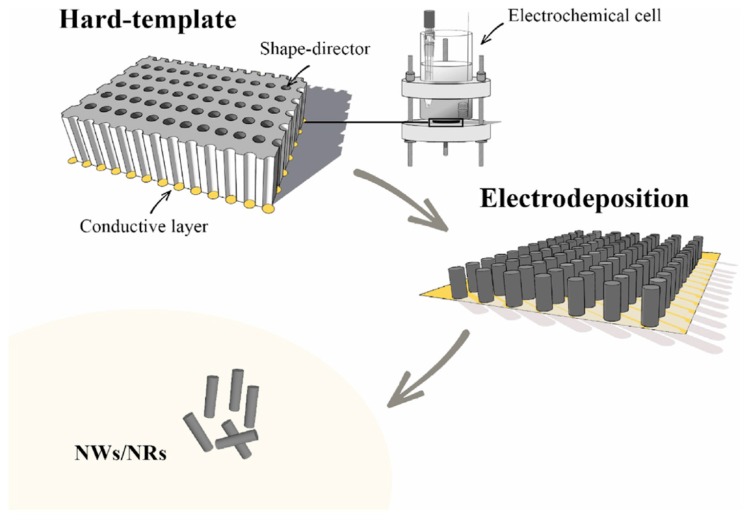
Schematic representation of NRs/NWs electrosynthesis using the hard-template approach. First, the hard template in which the nanochannels (shape director) are observed, equipped with a conductive layer in one of the two sides, is used as a working electrode in the electrodeposition process in a three-electrode cell. After electrodeposition the hard-template and/or conductive layer can be removed to obtain free-standing NRs/NWs. Image reproduced from [24] with permission.

For various applications, nanostructures in the form of rods (NRs) or wires (NWs) are unconventional substitutes for typical nanoparticles, due to their high aspect ratio and very low mass of material, that are fundamental properties in the field of (bio)sensing. Sensors based on 1D nanostructures display a high sensitivity due to the presence of numerous adsorption sites. Electrochemical deposition of NRs and NWs generally needs the use of templates, such as porous membranes to induce the shape of the 1D structures. The template needs to be conductive on one side so that it can work as a cathode. To this aim a nanometric layer of gold/graphite or other conducting materials is commonly used. By checking potential or current density to be applied, the reduction reaction can occur so inducing the growth of the nanostructures inside the templates [23]. The template which is most frequently used is anodized alumina, but other materials like radiation track-etched polycarbonate membranes or zeolites, porous silicon, mica, carbon nanotubes, and nanochannels patterned by photolithography have been also reported. After removal of the template, which is performed by dissolution with an organic solvent in case of polymeric membranes or with a strongly basic solution in the case of alumina, the NRs/NWs are released (Figure 3). The advantage of the template approach is linked to the production of nanomaterials with a perfect shape control and orientation [24].

Besides the modification of the surface electrode with nanomaterials, the nanostructuration can be obtained also by surface nanopatterning. This approach exploits the formation of a thick oxide layer using a repetitive square-wave perturbing potential in 0.5 M H_2_SO_4_ solution or by anodizing the Au electrode at a constant potential of 2.44 V vs. SHE. Later, the oxide layer is submitted to electroreduction under either a slow potential sweep or a potential step, in the same acid solution [25]. The resulting nanopatterning produces rough electrodes with a large active surface area, which can be favorably exploited for the preparation of bioelectrodes with improved analytical performance [26].

### 2.3. Electrochemical Sensors Based on Au-NPs

The peculiar chemical and physical properties of metal nanomaterials make them extremely suitable to be employed as electrochemical sensors, mostly supported on the electrode [12,27]. Such modified electrodes generally display reduced overpotentials toward many redox reactions and even are able to turn into reversible reactions that appear irreversible at classical electrodes. Furthermore, the small sizes of the NPs causes a very big increase in current in respect to the same material in the macro form. Au-NPs based electrodes have proven to be highly performant sensors for heavy metal detection. Most of them are fabricated by electrochemical deposition. The literature reports that the modification of GC surface with Au-NPs offers further advantages such as the elimination of memory effect and coexisting ions interferences, and higher sensitivity for the determination of Hg(II) and As(III) if compared to the bulk Au electrode [28].

A sensitive method for the electrochemical detection of Hg(II) in real environmental samples has been reported, using a nanocomposite obtained by electrochemical deposition of Au-NPs on a reduced graphene oxide modified GCE. In addition, thymine-1-acetic acid which displays a high affinity to Hg^2+^ was covalently bound to Au-NPs using cysteamine. The sensor was able to detect mercury in the range of 10 ng/L–1.0 mg/L, and showed a very good selectivity for Hg(II) in respect to many other heavy metal ions. Furthermore, the developed device was easily reusable through a simple washing [29].

A GCE was modified with gold nanoparticles, obtained by potential cycling from −0.4 to +1.1 V, and used as the support for the simultaneous determination of arsenic and selenium in water, by anodic stripping. The presence of Au-NPs improved both the stripping current and the peak resolution. The deposition potential, pH and choice of electrolytes were optimized so that detection limits of 0.15 ppb for As(III) and 0.22 ppb for Se(IV) were obtained, and the modified electrode was successfully applied to the analysis of real water samples [30].

Pb(II), Cd(II), and Cu(II) were detected simultaneously with a GCE modified with small and size-controlled Au-NPs electrodeposited on carbon nanofibers. The hybrid modified electrode could determine the three heavy metals at a concentration lower than 0.1 μM [31].

GCEs have been modified with Au-NPs of different sizes (from 3.5 to 21.5 nm) and shape (spherical and platelets) to investigate the cyclic voltammetric response of various compounds of biological interest. The results demonstrated a dependence of the electrochemical response on the dimension and the shape of Au-NPs that could be exploited to fabricate chemical sensors with enhanced selectivity [3].

Hydrogen peroxide determination in biological systems is important in clinical diagnosis, since a lot of oxidase enzymes produce H_2_O_2_ as a co-product, and in the industrial field it is also a fundamental intermediate reagent in the textile, paper, food, and pharmaceutical industries.

Metal nanomaterials have gained great attention also for the development of non-enzymatic electrochemical sensors for H_2_O_2_ because of their high stability compared to enzymatic sensors [32]. As far as nano-gold is concerned, a drawback that has been pointed out is related to the aggregation of Au-NPs which limits their performance, particularly in terms of detection limit which cannot reach low values. To overcome this problem an electrochemical sensor for H_2_O_2_ has been fabricated by electrodepositing Au-NPs (average size = 12 nm) on an ITO support previously coated with a Co, Mn-based layered double hydroxide. Electrochemical characterizations proved that the presence of the LDH support significantly improved the voltammetric response to H_2_O_2_ [33]. At a working voltage of +0.55 V (vs. Ag/AgCl), the sensor displayed a wide linear range (0.1 μM to 1.27 mM), low detection limit (0.06 μM) and high sensitivity. All these properties are better than the ones displayed by most previously described electrodes modified with AuNP-based composites.

3D Au nanodendrites were produced on a Pt support by electrodeposition, using a gas bubble dynamic template procedure in the presence of iodide ions to prevent agglomeration [34]. The modified Pt electrodes were applied to As(III) detection at ultralow concentration (0.1 ppb) in 0.2 M HCl solution.

### 2.4. Electrochemical Sensors Based on Nanoporous Au

Porous noble metal nanostructures for electrochemical sensing are appealing due to their higher specific surface areas and larger pore volumes, which facilitate both electron and mass transfer. Therefore, they allow the fabrication of sensors with very high sensitivities and very low detection limits [9].

3D nanoporous Au films, consisting of interconnected filaments and nanopores, were obtained on a Ni foam by the dealloying method using a simple two-step procedure. First, the Au-Sn alloy film was galvanostatically electrodeposited on the Ni foam and then the coated support was immersed into 5 M NaOH and 1 M H_2_O_2_ solution for 3 days. The resulting electrodes displayed an increased activity for the electroreduction of H_2_O_2_ in acidic solution, and were stable over time. In particular, chronoamperometric responses were continuously recorded for 6400 s using the same electrode, soaked in 0.5 M H_2_SO_4_ and 1.5 mM H_2_O_2_ and the currents were still constant [35].

A nanoporous gold (NPG) microelectrode, again produced by the electrochemical alloying/dealloying method, was employed to simultaneously quantitate hydrazine, sulfite and nitrite by electrochemical oxidation, displaying not only good sensitivity but also improved selectivity and stability. The three analytes showed well separated peaks centered at 0.05, 0.34, and 0.76 V, respectively, and the electrode was able to determine hydrazine, sulfite and nitrite within a wide concentration range (from 5.0 to 4000 μM) with low limits of detection (order of magnitude 10^−7^ M) [36]. The NPG microelectrode possessed high stability and selectivity. The performance of the sensor was also demonstrated with real samples such as water, wine, apple cider beer and beef, so to check its use for applications in food safety and quality control.

An unconventional very thin (100 nm) nanoporous Au leaf produced by dealloying has been proposed which shows an excellent electrocatalytic activity toward nitrite oxidation [37]. The response of nitrite ions was found to be independent of pH in the range from 4.5 to 8.0, which is a result significantly different from that relevant to a planar gold electrode, taken into account for comparison purposes. The nitrite determination was performed by chronoamperometry and the resulting calibration graph was linear from 1 µM to 1 mM, with a LOD equal to 1 µM. The nano electrode displayed good selectivity since it did not exhibit interference from commonly present compounds, such as sodium sulfate, potassium chloride, ammonium nitrate, glucose and ethanol. The authors attributed the remarkable improvement in the catalytic current to the increased surface area, the very easy transport of small compounds within the nanostructure constituted of only two or three layers of pore channels and Au filaments, and the presence of many low-coordinated surface gold sites.

The non-enzymatic sensing of glucose based on the direct electrochemical oxidation is a simple and cheap approach. Noble metals have been considered the most promising electrocatalysts for such a determination. The greatest advantage of amperometric non-enzymatic glucose sensors with respect to biosensors is related to the fact that the latter display a poor long-term stability due to the essential nature of enzymes. The major drawbacks of the direct electrochemical oxidation of glucose are the slow kinetics, the adsorption of the intermediates on the electrode which causes fouling, and the poor selectivity towards any other endogenous species that can be oxidized in the same potential window [38].

The discovery that nano-gold has catalytic activity for many important reactions offers an alternative strategy for the oxidation of d-glucose to d-gluconic acid with molecular oxygen, and it has been demonstrated that nano-gold exhibits an intrinsic activity similar to that of glucose oxidase enzyme [39]. By applying a square-wave potential program, in the presence of surfactant, Pt−Au alloy films were realized with a controlled composition which displayed a very high electrocatalytic activity for glucose oxidation, due to the favorable factors resulting from the mesoporous structure of the sensing material and the Au presence. The composition with the best performances was Pt51Au49 alloy film. Using this material the response to glucose was linear up to 11 mM and the detection limit resulted 6.0 μM [40].

GCEs modified with Au nanocages, which display a very large surface-to-volume ratio, have been proposed to develop sensors able to sensitively determine glucose by electrocatalytic oxidation. Taking into account that ascorbic and uric acids could interfere in the amperometric detection of glucose an operative potential of 0 V was chosen and the calibration curve resulted linear from 0.2 to 13.4 mM with a LOD of 5 μM [41]. Uniform Au nanocages could be prepared by using electrodeposition with Ag nanocubes as templates as already reported by reported by Torimoto et al. [42].

### 2.5. Biosensors

Biosensing is another important research field where metal NPs have been largely utilized in the last few years, especially when supported on conductive materials [27]. The NPs act as electrical wires to establish a direct electrical communication between the biocatalysts and the electrode which is generally hampered by the thick insulating protein shell that surrounds the enzymes’ active centres. The development of enzymatic biosensors based on the direct electron transfer (DET) has recently attracted great interest. Au-NPs functionalized with the cofactor flavin adenine dinucleotide (FAD) and supported on a gold macroelectrode have been reconstituted with the apo-glucose oxidase exhibiting excellent electron transfer properties, and the system was found to be a very useful glucose detection device [43].

**Figure 4:**
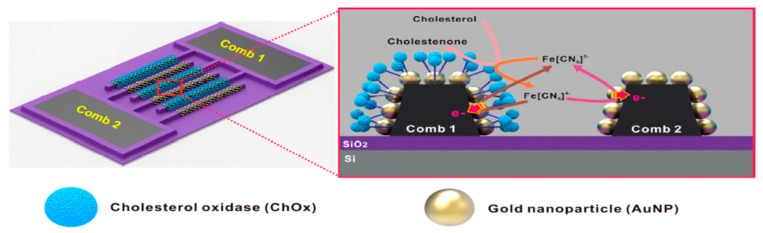
Schematic diagram of the sensing principle based on the redox cycling of [Fe(CN)_6_]^3−^/[Fe(CN)_6_]^4−^ between enzyme and electrode surfaces at nano-sized gold nanoparticle (Au-NPs)/carbon interdigitated electrodes (IDEs), selectively functionalized with cholesterol oxidase (ChOx); Working electrodes (comb 1: enzyme-functionalized Au-NPs/carbon comb, comb 2: non-functionalized Au-NPs/carbon comb. Image reproduced from [44] with permission.

In order to develop a cholesterol biosensor, Au-NPs were selectively electrodeposited on nano-sized carbon interdigitated electrodes by tuning the step-potential and time period, and cholesterol oxidase was immobilized through the electrochemical reduction of the diazonium cation. The biosensor displayed high selectivity toward cholesterol and high sensitivity which was ensured by the use of the efficient redox mediators, ferricyanide and ferrocyanide (Figure 4). In particular, the sensing range was wide (0.005–10 mM) and the LOD resulted ~1.28 μM [44].

Combining electrochemical deposition with the Langmuir−Blodgett technique highly ordered macroporous Au was synthesized and used to fabricate an amperometric biosensor based on glucose dehydrogenase and (4-carboxy-2,5,7-trinitro-9-fluorenylidene) malononitrile as a redox mediator. The electrocatalytic current recorded for glucose oxidation was one order of magnitude higher than the one recorded at a non-porous Au electrode [45].

3D ordered macroporous Au film was also employed to develop a novel label-free immunosensor for the determination of C-reactive protein (CRP) using electrochemical impedance spectroscopy (EIS) [46]. The film consisted of interconnected Au-NPs which exhibited a large surface area (almost 15 times greater than the one of the flat Au electrode) for the immobilization of the protein and displayed linearly increasing impedance values with CRP concentration (over a linear range from 0.1 to 20 ng·mL^−1^).

NPG electrodes were also constructed to increase the active surface area in order to produce advanced electrochemical platforms for DNA detection. As an example, such sensors were used to amplify the DNA signal and could detect single base mismatches and complementary target DNA. Ferrocene carboxylic acid (FCA) was covalently attached on the top of probe DNA, which was hybridized with target DNA, and the electrochemical oxidation signal of FCA was recorded as the analytical signal [47].

An electrochemical DNA biosensor based on NPG electrodes, prepared with repetitive square-wave oxidation/reduction cycles, was constructed for the determination of promyelocytic leukemia/retinoic acid receptor α fusion genes in acute promyelocytic leukemia, using methylene blue (MB) as the electroactive probe [48]. The active surface area of the NPG electrode was about one order of magnitude greater than the one of a bare flat counterpart. The MB response, studied by differential pulse voltammetry, decreased when the probe was hybridized with target DNA. The DNA biosensor was specific for the complementary strand and the obtained response was linear within a concentration range from 60 pM to 220 pM, with a LOD of 6.7 pM.

Nanostructured Au deriving from the dealloying technique was utilized to construct an electrochemical biosensor based on glucose oxidase which displayed a very stable enzyme immobilization and optimum performance for glucose sensing thanks to the uniformly distributed Au nanostructures [49].

As far as the surface nanopatterning is concerned, a lactate enzyme biosensor was developed exploiting a nanostructured rough gold surface obtained by electroreduction of a thick oxide layer, previously pre-formed in acid solution [25]. The nanopatterning process generates a very high active area that allows for a high lactate oxidase loading and decreases the charge transfer resistance, as verified from EIS experiments. As a consequence, the biosensor shows a wider linear range concentration (up to 1.2 mM) and higher sensitivity for lactate determination, if compared to a polycrystalline gold electrode [50].

The optimal performances displayed by the lactate biosensor suggested to other researchers that rough Au electrode surfaces can be considered as promising electrochemical transducers for the production of other bioanalytical platforms. In fact, DNA biosensors based on nanostructured Au electrodes have been fabricated for the detection of synthetic short DNA sequences [47], typical genes fragments and bacterial DNA [26].

Nanopatterned Au was used to develop a novel genosensor for the detection of a characteristic gene (lacZ gene) of the Enterobacteriaceae family which was usefully applied to detect PCR amplified real samples, using a simple sample pre-treatment. A synthetic 25-mer DNA capture probe, modified at the 5′ end with an alkylthiol, able to hybridize with a specific sequence of lacZ gene, was assembled on the rough Au surface, and the extent of hybridization was electrochemically recorded by using two different complexes of aminoruthenium (III). The developed genosensor showed a remarkable long-term stability, a wide linear range for target concentrations, and a higher sensitivity than a non- nanostructured biosensor [26].

## 3. LDHs and Other Inorganic Materials

### 3.1. Electrodeposition

The first authors to report the electrochemical synthesis of LDHs were Indira and Kamath in 1994 [51,52] who described the synthesis of bulk samples of LDHs containing Co(II) or Ni(II) and Al(III) in the bivalent/trivalent metal ratio equal to 3, by cathodic reduction of nitrate ions and water to generate the basic environment necessary for LDH precipitation. The one-step process consisted of a galvanostatic method, applying a fixed current density for 4 h, in a divided electrochemical cell with a platinum flag (surface area 3 cm^2^) cathode.

When a cathodic polarization is applied to the working electrode in an aqueous solution containing nitrates and a bivalent and a trivalent metal of radius compatible with that of a LDH structure, the following Reactions (1)–(7) occur, with the overall effect of increasing the pH close to the electrode surface making it suitable for the occurrence of the LDH precipitation:

H^+^ + e^−^ → H_ads_ (1)

2H^+^ + 2 e^−^ → H_2_ (2)

NO_3_^−^ + 2 H^+^ + 2 e^−^ → NO_2_^−^ +H_2_O (3)

NO_3_^−^ + 10 H^+^ + 8 e^−^ → NH_4_^+^ + 3 H_2_O (4)

2 H_2_O + 2 e^−^ → H_2_ + 2 OH^−^ (5)

NO_3_^−^ + H_2_O + 2 e^−^ → NO_2_^−^ + 2 OH^−^ (6)

NO_3_^−^ + 7 H_2_O + 8 e^−^ → NH_4_^+^ + 10 OH^−^ (7)

For sensing applications it is desirable to produce thin films well adherent to the electrode surface, thus, the procedure proposed by Kamath and co-workers had to be adapted and optimized to provide films of controlled thickness, homogeneously coating the electrode surface.

To this aim, Tonelli’s research group conducted an extensive study to optimize the electrochemical deposition of a Ni/Al LDH on Pt electrodes [53]. The procedure is based on the potentiostatic reduction of nitrate ions for a very short time (a full electrode coverage can be achieved within 60 s), the film thickness being easily tunable by modifying the applied potential and electrodeposition time. Moreover, since peculiar features of a chemical sensor are the repeatability and reproducibility of its response, the electrode cleaning procedure before the electrodeposition step was thoroughly investigated with the aim of enhancing the mechanical adhesion of the coating to Pt surface [5]. The results of Tonelli’s group demonstrated that an electrochemical pretreatment of the Pt surface in sulphuric acid (consisting of 250 CV cycles between 0.20 and +1.30 V vs. SCE in 0.1 M H_2_SO_4_, at a scan rate of 1 V·s^−1^ and a subsequent application of a cathodic potential of −0.90 V for 300 s in 1 M H_2_SO_4_), was the best one to obtain an improved performance of the LDH films.

**Figure 5:**
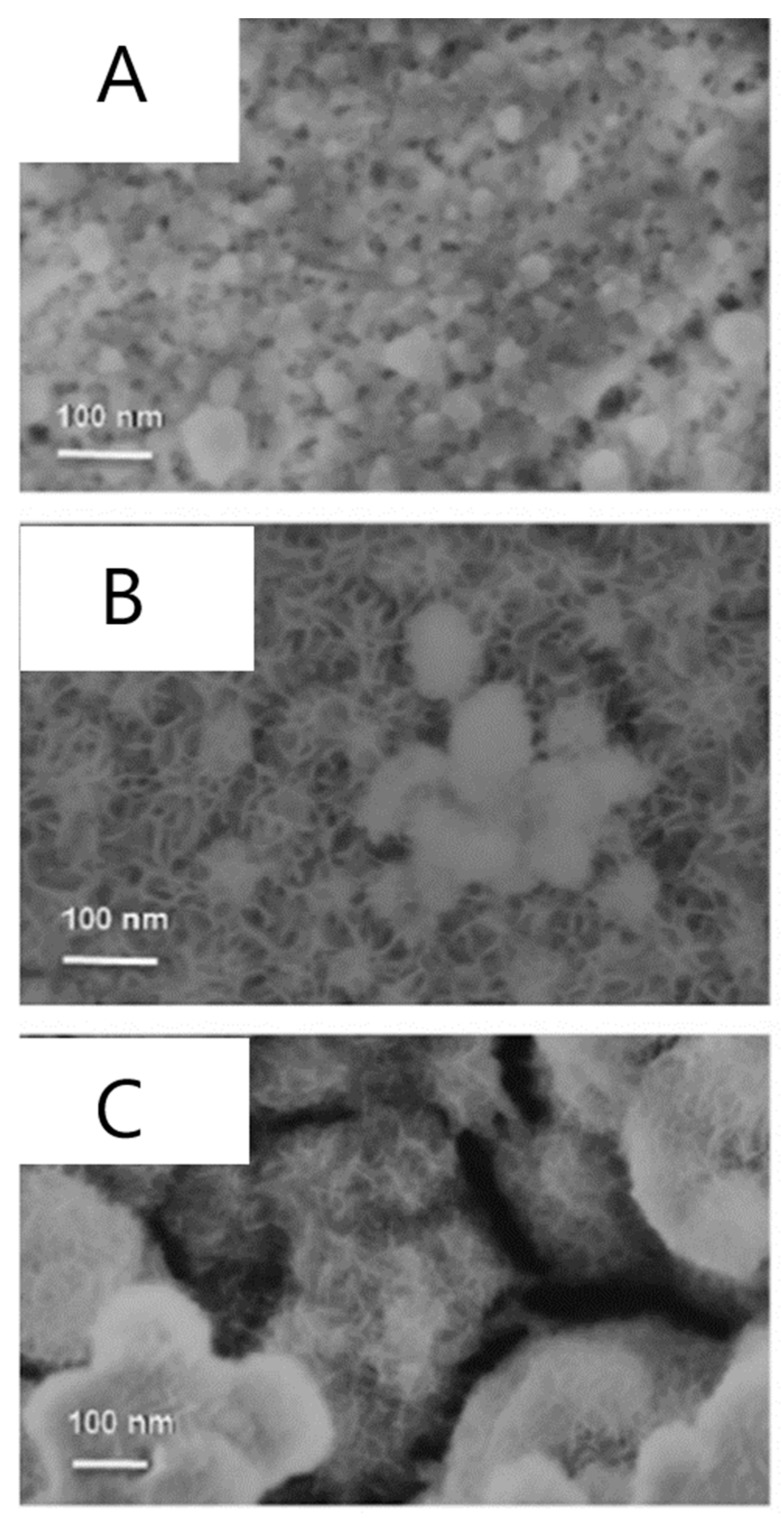
SEM images of LDH films coating a Pt substrate, prepared by potentiostatic electrochemical deposition at −0.9V vs. SCE for 60 s (**A**), 120s (**B**), 200 s (**C**). Image reproduced from [53]. Copyright (2007) American Chemical Society.

The textural properties of the LDH films prepared by potentiostatic electrodeposition were dependent on the duration of the potential pulse. Figure 5 shows the SEM characterization of Ni/Al LDH coatings coming from pulses of different length, i.e., 60 (A), 100 (B) and 200 s (C). At 60 s, a dense and homogeneous membrane made of nanoparticles with an average size lower than 50 nm, and connected in a gel-like manner was obtained. When the electrodeposition lasted longer, nanoparticles having a larger mean dimension were formed, still retaining the sand rose morphology typical of LDHs.

Also the thickness of the LDH films clearly depended on the electrodeposition time: generally films of about 150 nm were obtained for a deposition time of 60 s, and this thickness was the most suitable to obtain LDH films suitable for sensing applications.

### 3.2. Electrochemical Sensors Based on Redox Active LDHs in Basic Solution

Electrodes coated with Ni/Al and Co/Al LDH thin films can be used as chemical sensors for a wide range of analytes thanks to the capability of the bivalent metal (Me = Ni or Co) to act as a redox mediator following the reaction scheme:

Me(II)LDH + OH^−^ ⇌ Me(III)-OHLDH + e^−^ (8)

Me(III)LDH + reduced analyte → oxidized analyte + Me(II)LDH(9)

The reaction needs a basic environment to occur effectively (OH^−^ ions that diffuse inside/outside the LDH layers play a key role in the electrocatalytic process) and this fact limits the pH range of Ni or Co based LDH thin films applications, being the required pH higher than 10.

The nature of the bivalent metal and its redox potential affect the nature of detectable analytes: actually, Ni has an electrocatalytic activity for the oxidation of alcohols, polyhydric compounds and amines [54,55]. The substitution of Ni with Co induces a selectivity in the electrooxidation of molecules containing hydroxyl groups, due to the lower redox potential of Co, and thus Co-based LDHs do not display electrocatalytic activity towards monohydric compounds. Consequently, only molecules containing more than one hydroxyl functional group (e.g., glycerol, monosaccharides, polysaccharides, salicylic acid, etc.) can be oxidized at electrodes modified with Co-based LDHs [56,57].

**Figure 6:**
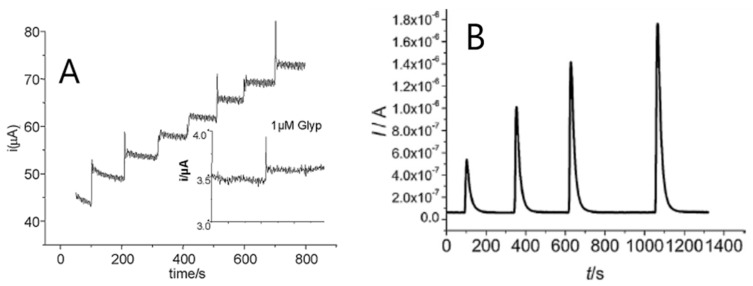
Examples of I vs. t curve obtained (**A**): at a Ni/Al LDH coated Pt electrode in batch for seven successive additions of 0.125 mM Glyp, inset shows the LOD of Glyp (E_appl_ = +0.49 V vs. SCE) in 0.1 M NaOH; (**B**): at a Co/Al LDH coated Pt electrode in FIA, by injecting fructose solutions at different concentrations (1.0 × 10^−4^, 2.0 × 10^−4^, 3.0 × 10^−4^ and 4.0 × 10^−4^ M). Eluent: 0.01 M NaOH containing 0.1 M KNO_3_, flow rate: 1 mL min^−1^; E_app_: + 0.50 V vs. Ag/AgCl. Images reproduced from [59,60] with permission.

Sensors exploiting thin films of LDHs have been proven very useful also in the detection of simple analytes that usually cause problems of fouling at the electrode surface, such as phenol [5], or in the detection of more complex compounds of environmental concern, such glyphosate (Glyp) and gluphosinate (Figure 6A) [54]. Their response is very stable, lifetime being typically of about 15 days and the mechanical stability is so good that the sensors can effectively operate both in batch and in flow conditions (Figure 6B).

All these results point out the role of the bivalent metal in determining the electrocatalytic features of the LDH thin films, but recent studies have shown that the presence of a redox active trivalent metal, such as iron, in the brucite structure causes an enhanced performance of the modified electrodes due to a higher number of Me(II) electrochemically active sites [58].

All the modified electrodes described so far exploit an electrocatalytic oxidative process occurring in the anodic potential range. A different transduction mechanism was proposed by Qiao et al. for the detection of antracene in 1 M KOH solution [61]. The authors used the electrodeposition approach to prepare Cd/Al-LDHs-coated GC electrodes. The proposed sensor was based on the redox reaction Cd(II)+2e^−^ → Cd which requires the loss of OH^−^ ions from the LDH structure to maintain neutrality. The presence of anthracene in the solution hindered such a loss since the organic compound adsorbed on the surface of the LDH, thus influencing the Cd peak signal. This property allowed the detection of the analyte in real samples of cloud-rain water [61].

### 3.3. Analytical Applications of LDHs in Non Basic Environment

Besides the analytical applications of the above described LDHs, materials not containing a redox active metal can also be employed for the development of sensors. As an example, the capability of the LDH to preconcentrate the analyte can be exploited to produce amperometric sensors displaying very low LODs. Li et al. developed a dihydroxy-benzene sensor by electrochemical potentiostatic deposition of a Zn/Al LDH film on glassy carbon electrode (LDH/GCE). The LDH/GCE was used for the sensitive and simultaneous determination of catechol (CA), and hydroquinone (HQ), in the presence of resorcinol (RE), through differential pulse voltammetry (DPV) [62].

Under the optimized conditions, the DPV response of the modified electrode to CA (or HQ) showed a linear concentration range from 0.6 μM to 6.0 mM (or from 3.2 μM to 2.4 mM) and the calculated limit of detection was 0.1 μM (or 1.0 μM).

GCEs coated with a Zn/Al LDH synthesized by electrodeposition were also employed for the detection of phenolic, gallic and caffeic acids. The results indicated that after the electrode modification, the oxidation currents of the analytes were greatly enhanced due to the preconcentration performed by the LDH [63].

Among the applications of LDHs as electrode modifiers to fabricate devices able to operate in a non-basic environment the use of LDHs as a matrix to support enzymes is noteworthy. The approach to immobilize the glucose oxidase enzyme during the electrodeposition step of a Ni based LDH was proposed by Tonelli et al. obtaining in a very reproducible and fast way a glucose biosensor [64]. The same procedure was also employed to immobilize lactate oxidase, with the aim of realizing a lactate biosensor [65].

### 3.4. Composite Systems Layered Double Hydroxides-Metal NPs

Metal nanoparticles and LDHs can be coupled to obtain, by electrodeposition, composite materials able to confer an enhanced performance to the device. Xu et al. prepared a nanocomposite made of Au-NPs and Co/Mn LDH on an ITO electrode to fabricate a hydrogen peroxide sensor that displayed good stability and optimum electrocatalytic activity toward H_2_O_2_ oxidation. The excellent performance of the sensor can be attributed to the multiple synergetic effects between Au-NPs and the Co/Mn LDH support that reduce the size of the nanoparticles and improve the conductivity of the composite material [33]. A sensor for H_2_O_2_ was also developed exploiting a composite material made of Ag dendrites and LDH, obtained by electrodeposition of the nanostructures on a GCE previously coated with a Mg/Al LDH [66].

**Figure 7:**
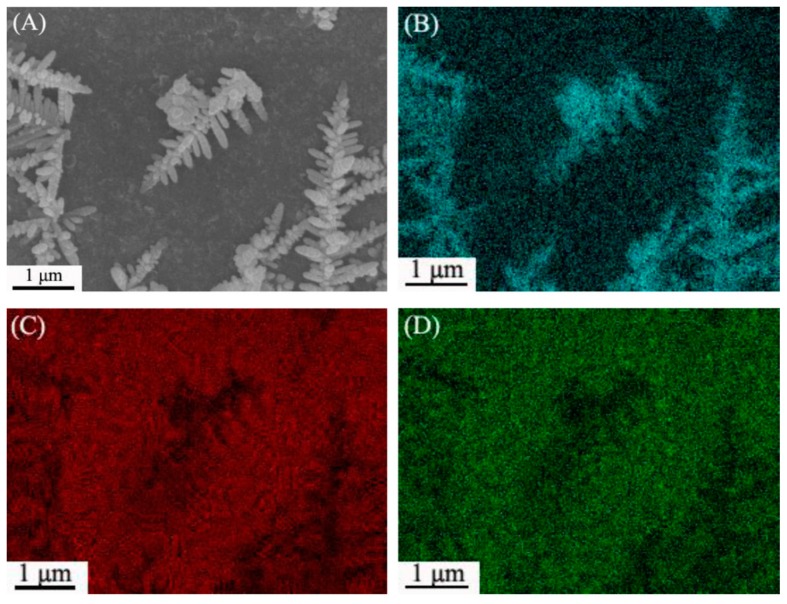

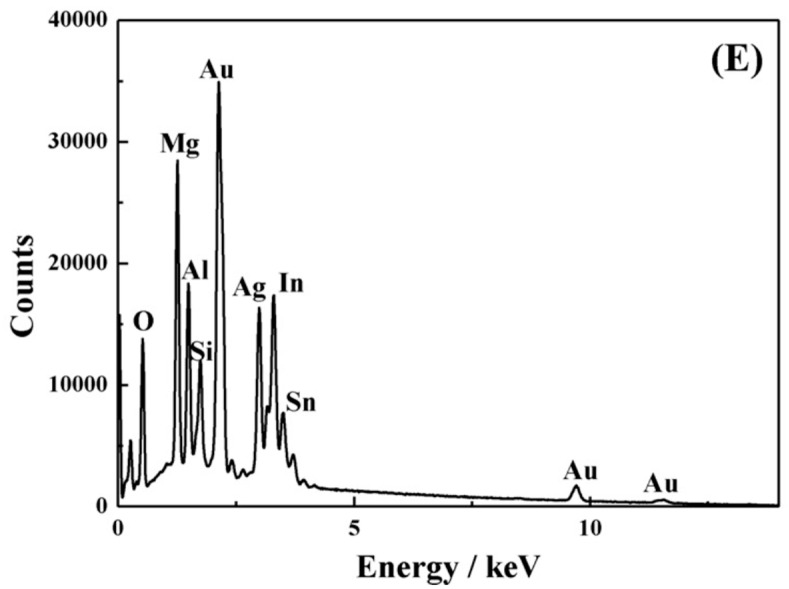
(**A**) SEM image of Ag/LDH/ITO and its corresponding elemental mappings for (**B**) Ag, (**C**) Mg and (**D**) Al. (**E**) the corresponding EDX spectrum taken from the whole area of (**A**). Images reproduced from [66] with permission.

The authors demonstrated that the presence of the LDH significantly enhanced the electrocatalytic performance of the sensor. The Ag-NPs/LDH coated electrode showed a linear response in a range from 10 μM to 20 mM and a LOD of 2.2 μM, whereas in the absence of the LDH the sensor showed a limited linearity range (from 0.1 to 10 mM) and a higher LOD (46 μM). The LDH substrate induced the formation of more Ag dentrites with smaller size, resulting in a higher surface area which, in turns, leads to a higher electrocatalytic activity. The SEM characterization of the modified electrode using ITO glass as a substrate is shown in Figure 7.

Besides the application for H_2_O_2_ detection, nanocomposites made of metal NPs/LDH can be utilized in other fields. Cui et al. developed a sensor for nitrite based on the electrodeposition of Au-NPs on a GCE modified with a copper calcined layered double hydroxide (Cu-CLDH). Electrochemical experiments showed that Au-NPs/CLDH composite film exhibited excellent electrocatalytic activity for nitrite oxidation due to the synergistic effect of the Cu-CLDH and Au-NPs. The superior electrocatalytic response to nitrite was mainly attributed to the large surface area, minimized diffusion resistance, and enhanced electron transfer of the Cu-CLDH and Au-NPs composite film [67].

GC or graphite electrodes modified by electrodeposition of Pt-NPs on which a Ni/Al LDH was electrochemically deposited also showed enhanced performance for glucose and ethanol detection; in particular the presence of Pt-NPs allowed to achieve a wider linearity range. When the support was GC the upper limit of concentration for ethanol determination was 65 mM [68].

A similar composite was proposed by Gong et al., who intercalated Pt nanoparticles inside the interlayer of a Ni based LDH to develop a sensor for organophosphate pesticides (OPs) detection. The resulting composite matrix facilitated the preconcentration of methyl parathion (MP), chosen as a model compound of OPs, through solid-phase extraction and made possible a sensitive stripping determination by square wave voltammetry, with a LOD of 0.6 ng mL^−1^ [69].

**Figure 8:**
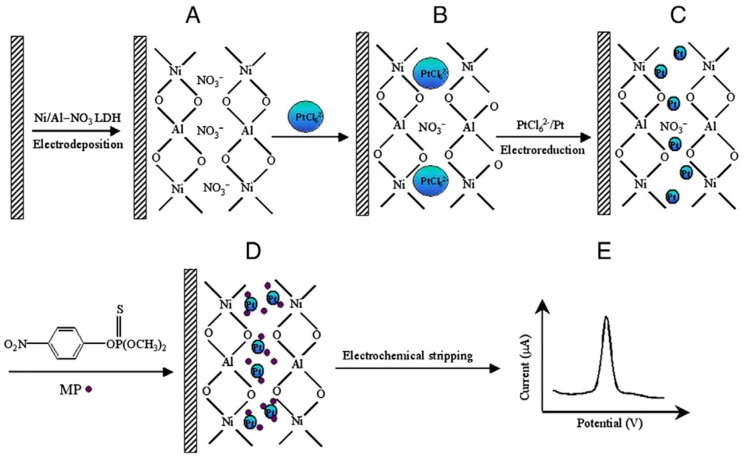
Schematic illustration for electrochemical sensing MP: (**A**) electrodeposition of a Ni/Al–NO_3_–LDH film onto a GCE surface; (**B**) subsequent exchange of PtCl_6_^2−^ with NO_3_^−^ anions; (**C**) followed by electrochemical reduction to form the assembly of NanoPt-LDH onto GCE; (**D**) MP intercalated into the interlayer space of NanoPt-LDH/GCE; (**E**) electrochemical stripping detection of MP. Image reproduced from [69] with permission.

The authors proposed a new electrochemical sensing protocol (Figure 8) which involves the one-step electrosynthesis of a thin Ni/Al-LDH film onto a GC electrode (A), subsequent incoming of PtCl_6_^2−^ (B), followed by electrochemical reduction to form the assembly of NanoPt and Ni/Al-LDH(C), then the intercalation of MP into the interlayer space (D), and finally the electrochemical stripping detection of the adsorbed MP (E).

### 3.5. Metal Oxide Nanoparticles

Several transition metal oxides NPs can be used as electrode modifiers to build electrochemical sensors. Among the most used transition metals are iron, copper, cobalt, nickel, manganese, titanium, silver, vanadium, zirconium, zinc, and tungsten. Metal oxide nanoparticles can be prepared by means of different methods and a comprehensive review has been recently published [70]. Among them, electrochemical deposition plays an important role, mainly to synthesize copper or nickel oxide NPs.

The most extensively used metal oxide nanostructures for electrochemical sensing applications are CuO and Cu_2_O. Different morphologies have been obtained using chemical or electrochemical protocols [71,72,73,74] on various electrode materials, mainly carbon-based, such as graphene, carbon nanotubes or carbon fibers, since these supports have been demonstrated to enhance the charge transfer and, consequently, the device performance [75,76,77]. The most important application in the field of electrochemical sensing of electrochemically deposited copper oxides is glucose oxidation, in basic solution, which exploits the capability of copper to act as a redox mediator.

**Figure 9:**
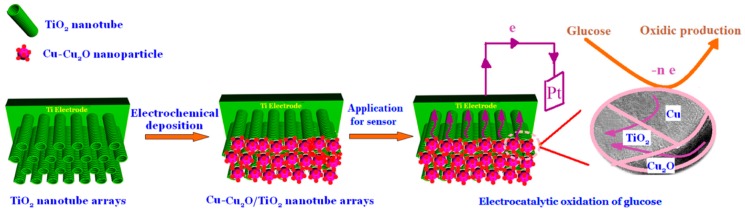
A schematic illustration of the preparation of the Cu−Cu_2_O/TiO_2_ nanotube array/Ti electrode and the electron transfer through the Cu−Cu_2_O/TiO_2_ nanotube array interface for the electrocatalytic oxidation of glucose Image reproduced from [78]. Copyright (2015) American Chemical Society.

As an example, Yang et al. prepared a non-enzymatic glucose sensor based on a composite Cu_2_O/TiO_2_ [78]. The first step of the electrode preparation was the electrodeposition, by anodic oxidation, of helical TiO_2_ nanotubes array with a diameter of about 105 nm, followed by a second step in which a layer of a Cu and Cu_2_O mixture was deposited, with an approximate thickness of several hundreds of nanometers. The authors obtained an electrode with excellent electrocatalytic activity toward glucose oxidation (Figure 9). The linearity between the response current and the glucose concentration was demonstrated in the range from 0.1 to 2.5 mM with a sensitivity of 4895 μA cm^−2^ mM^−1^. Such a high sensitivity was attributed to the synergistic effect of the small Cu−Cu_2_O grain size and the large surface area of the helical TiO_2_ nanotube arrays as well as to the fast electron transfer.

Cu_2_O nanoparticles of controlled size were also prepared by electrodeposition in the presence of ethylenediamine (EDA) in the electrolytic bath. Through this procedure, a precise control of the size and morphology was demonstrated, obtaining Cu_2_O NPs from 54 to 966 nm [79].

Among metal oxides, Ni oxide modified electrodes are also very interesting for chemical sensing, and they exhibit a remarkably high catalytic activity for glucose oxidation due to the formation of the Ni(OH)_2_/NiOOH redox couple in alkaline medium, involving an electrochemical process similar to that described for LDHs. Recently, a number of Ni oxide-based glucose biosensors has been investigated [80,81,82,83,84,85,86]. Actually, to improve the performance of the sensors, many fabrication processes, such as electrodeposition, have been investigated as well as composites with graphene and/or polymers, and alloying with other metals. Electrodeposition is particularly attractive and the process can be simply controlled by adjusting the applied current, scanning potential window, number of cycles and duration of the process, but the low sensitivity and narrow linear range are the major drawbacks for the electrodes modified with electrodeposited Ni oxide or hydroxide [80,85]. To overcome this problem, recently, Ni/NiO core–shell NPs were obtained on a GCE using a simple potentiodynamic method. The Ni/NiO–GCEs exhibited a high sensitivity and selectivity for the detection of glucose in a wide concentration range, from 2 μM to 14 mM and a very low detection limit of 0.4 μM [87].

NiO nanoparticles electrochemically synthesized on multi-walled carbon nanotubes (MWCNTs) were also used for the modification of glassy carbon electrodes to achieve lactose detection in NaOH. The authors used a pulsed potential electrodeposition process to accumulate nickel oxide (NiO) on the nanotubes and the NiO particles size was controlled by the number of potential pulses, becoming larger if the pulses increased [88].

Liu et al. [89] reported the simultaneous electrodeposition of nickel oxide NPs together with electrochemically reduced graphene oxide (ERGO) onto GC electrodes for the acetaminophen detection. The developed sensor showed excellent electrocatalytic activity toward the oxidation of acetaminophen owing to the synergic effect of Ni_2_O_3_/NiO particles and ERGO, the latter contributing to the increase of the accessible reactive area. The sensor displayed a low value of LOD (0.02 μM) and a wide linearity range for acetaminophen determination (from 0.04 μM to 100 μM) by DPV, and was satisfactorily applied to pharmaceutical products and urine samples.

## 4. Polymers

### 4.1. Electrosynthesis of Conductive Polymers

Electrosynthesis of conductive polymers is usually carried out by an oxidative polymerization of a suitable monomer. The most important polymers that can be produced with this approach belong to the classes of polythiophenes, polypyrroles and polyanilines. The cathodic polymerization is less used and the main applications concern the production of poly(p-phenylenevinylenes) and poly(p-xylylenes) which are used in the fabrication of light emitting devices. For these reasons, only the anodic polymerization is described in this review.

The overall reaction is:

(*n* + 2)HMH → HM(M)*n*MH^(*nx*)+^ + (2*n* + 2)H^+^ + (2*n* + 2 + *nx*)e^−^ (10)

where HMH and HM(M)*n*MH are the monomer and the polymer, respectively. The polymerization is induced by applying a potential to the electrode that is enough anodic to oxidize the monomer to form the radical cation. The coupling of radical cations followed by the elimination of two hydrogen ions leads to the formation of species with a higher molecular mass. Ideally, both the reaction of radical cations coupling and proton elimination are fast, but the experimental evidences suggest that the rate determining step is the H^+^ removing. For example, the addition of 1% water to acetonitrile significantly increases the rate of the polymerization by helping the extraction of one hydrogen ion because H_2_O acts as a base [90]. Since the higher is the number of the repetitive units in the polymer, the lower is the oxidation potential, the process can proceed by the formation of radical cations of the dimeric and oligomeric species that can react together to increase the molecular weight. The formation of polymer can be described by three stages. The first step is the production of soluble oligomers in the diffusion layer, mainly due to dimerization reactions. When the oligomers chain is long enough, the macromolecules are not anymore soluble in the solvent and, consequently, they precipitate on the electrode surface with nucleation and growth processes. Finally, the polymerization takes place in solid phase to produce longer chain molecules. It is worth noting that the polymeric species are more easily oxidizable than the monomeric one, therefore, a fraction of the charge that flows through the electrode is consumed to oxidize the polymer. For this reason, the polymer is produced in an oxidized form and *nx* electrons must be added to the reaction stoichiometry (reaction (10)). *x* represents also the fraction of charge for each repetitive unit in the polymer.

The polymerization can be performed either by exploiting a potentiostatic or a potentiodynamic approach. Both electropolymerization modalities offer advantages, but exhibit also disadvantages that are clearly described by Janákya and Rajeshwar [91].

The electrosynthesis of conductive polymers generally leads to a morphology characterized by a cauliflower-like structure. Such a kind of materials is widely used for sensing, but this review aims to describe the sensing applications obtained with more complex nanostructures. The most employed approach to obtain nanostructures exploits a template that is removed after electrochemical polymerization. A template can be used to obtain the desired morphology or to produce interaction sites for the analytes in order to have a molecularly imprinted polymer (MIP). Finally, also template-free syntheses have proposed in literature.

### 4.2. Electrochemical Polymerization of Insulating Polymers

The oxidative polymerization above described can be performed also for insulating polymers wherein the repetitive unit has an aromatic ring. The main difference with the synthesis of conductive polymers is due to the nature of the electrode modifier that cannot conduct current and, consequently, the film growth leads to the passivation of the electrode surface. On one hand, it is useless for the development of sensors that require a charge transfer to work. On the other hand, the insulating nature of the materials hinders the polymer growth and, consequently, this can be exploited to obtain film with a controlled thickness. For example, Gualandi and Tonelli have used this feature to produce reproducible polyphenol thin films which were used for the detection of OH radical by means of an aromatic hydroxylation [92]. This feature is widely utilized for the fabrication of MIP based sensors, because they require a very fine control of polymer thickness.

### 4.3. Analytical Applications of Nanostructured Conductive Polymers

Conductive polymers can be synthesized with a well-defined morphology by electrosynthesis [93] by the use of a template or by setting the electrosynthesis conditions. The general aim is the improvement of sensor performance by increasing the surface area. Nevertheless, this approach may hinder the charge transport in the polymer with a loss of performance.

Bai et al. [94] have thoroughly studied the effect of the parameters employed in template-free electrochemical polymerization of 3,3-bithiophene, 1,3,5-tri-(thiophen-2-yl)benzene, and tris(4-(thiophen-2-yl)phenyl)-amine on the morphology of the thin films. Nanovesicles, nanorods, nanocauliflowers and nanotubes can be obtained only by controlling finely the electrosynthesis conditions. The authors exploited these structures as active materials to develop a sensor for the detection of nitro-analytes by cyclic voltammetry. Similarly, Wu et al. [95] have studied the electrochemical polymerization of aniline in solutions containing different macromolecules, thus obtaining different nanostructures. The modified electrode has been used for hydrogen peroxide detection.

**Figure 10:**
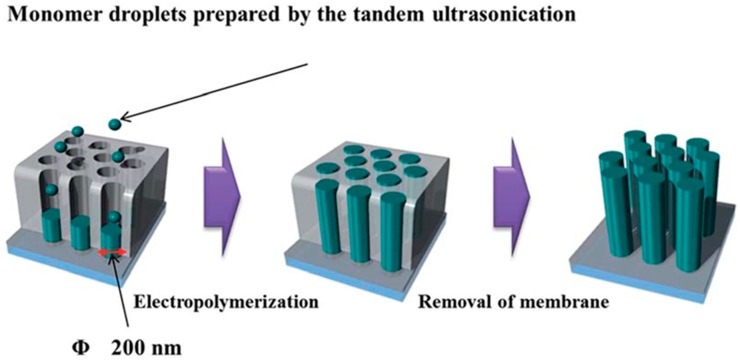
Sketch of preparation of PEDOT nanowires using template electrochemical polymerization. Image reproduced from [96] with permission.

Anodized aluminum oxide and track etched polycarbonate membranes are the templates usually employed for the preparation of nanotubes and nanowires. Figure 10 shows a sketch of the fabrication step to obtain PEDOT nanowires using a nanoporous alumina membrane [96]. Since these materials are insulating, a conductive layer, in the form of gold film, must be deposited on the template so that it acts as working electrode during the electropolymerization. The nanotubes morphology is obtained because the polymer is formed in the pores of the structure that is solubilized with a proper solvent after the synthesis.

Hajian et al. [97] used a porous alumina template to electrosynthesize polythiophene nanotubes, that were released by dissolving the template in 0.1 M NaOH solution. The nanotubes were suspended in ethanol and drop casted on a glassy carbon support. The modified electrode was used to electrochemically detect riboflavin. Salgado et al. [98] proposed PEDOT nanowires covered with polydopamine as electrode modifier for the detection of dopamine by cyclic voltammetry. The nanowires were produced by exploiting a silica template that was generated in situ on the Pt electrode. The nanowires derived from two steps electrochemical polymerization in order to obtain a core of PEDOT:PSS covered by a polydopamine layer, allowed reaching higher sensitivities than simple PEDOT nanowires.

For the above described sensors the signal transduction is based on the intrinsic electrocatalytic proprieties of the conductive polymer, but it is possible exploiting also the sensing features of an element that is co-deposited. Gokhale et al. [99] performed the electrosynthesis of PEDOT/nitrate reductase nanowires using a polycarbonate membrane and they utilized the chemically modified electrode for nitrate determination. Moreover, polypyrrole nanotubes have been electrodeposited together with glucose oxidase to fabricate an amperometric sensor with enhanced performance for glucose determination [100].

An intriguing approach is the use of conductive polymer nanotubes as components in an electronic circuit. Polypyrrole nanofibers for sensing were also produced by template-free electrodeposition on Si 100 in the presence of l-camphorsulfonic acid [101]. The interface between Si and nanofibers was exploited to produce a Schottky junction that can detect *m*-dihydroxybenzene thanks to the variation of electrical conductivity. The limit of detection was 1.51 mM, a value that is higher than the ones which can be obtained with the most common electrochemical techniques.

Conductive polymer nanotubes can be prepared by template electrodeposition and, after that, inserted between two electrodes to fabricate a chemoresistor sensor. Tolani et al. [102] used this approach to produce an immunosensor for human serum albumin detection in liquid samples. Moreover, polypyrrole and polyaniline have been used for H_2_ [103] and NH_3_ [104] sensing in gas matrix, respectively. A chemoresistive immunosensor has been even produced using only one polypyrrole nanowire [105]. Polypyrrole nanowire suspension was dispensed on 16 pairs of gold interdigitated electrodes with a separation of ~70 µm, while an alternating current field was applied between each pair of electrodes to induce an alternating current dielectrophoretic alignment. After completing the evaporation, excess nanowires were manually removed using a probe tip under a 1000× magnification optical microscope. The nanowires were anchored to the electrodes thanks to a gold electrodeposition that incorporates their extremities. After the modification with cancer antigens (CA 125), the sensor could detect cancer biomarkers in human blood samples.

**Figure 11:**
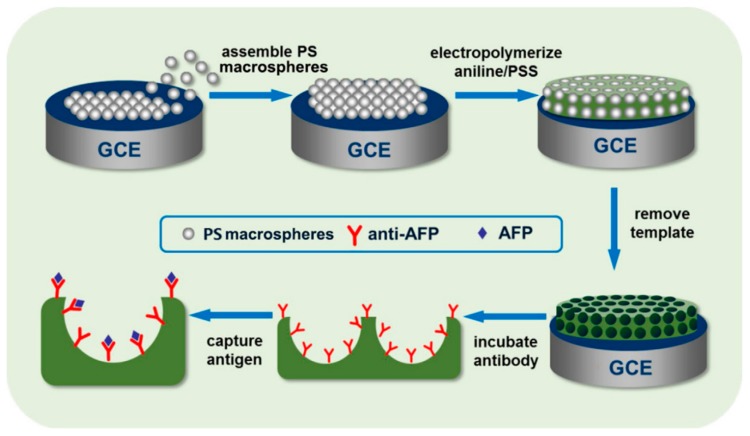
Sketch of fabrication of an immunosensor by using beads template. Image reproduced from [106] with permission.

Nanonetworks of conductive polymers can be obtained using a suitable template. A network of polyaniline was obtained by using polystyrene beads deposited on the electrode surface. The porous substrate was modified with a suitable antibody to produce an immunosensor for alpha-fetoprotein [106] (Figure 11). Nanonetworks can be also produced by exploiting the natural oxygen evolution that takes place at the anodic potentials applied for the electrochemical synthesis. The polymer is deposited between the bubbles and a porous structure is formed. Ma et al. have utilized this approach to obtain an electrode modified with a polypyrrole network functionalized with glucose oxidase for amperometric sensing of glucose [107]. Moreover, a methanol gas sensor has been fabricated by exploiting a nanostructured polypyrrole film obtained by electrochemical polymerization on interdigital electrodes, in the presence of perchlorate as dopant [108].

### 4.4. Molecularly Imprinted Polymers

**Figure 12:**
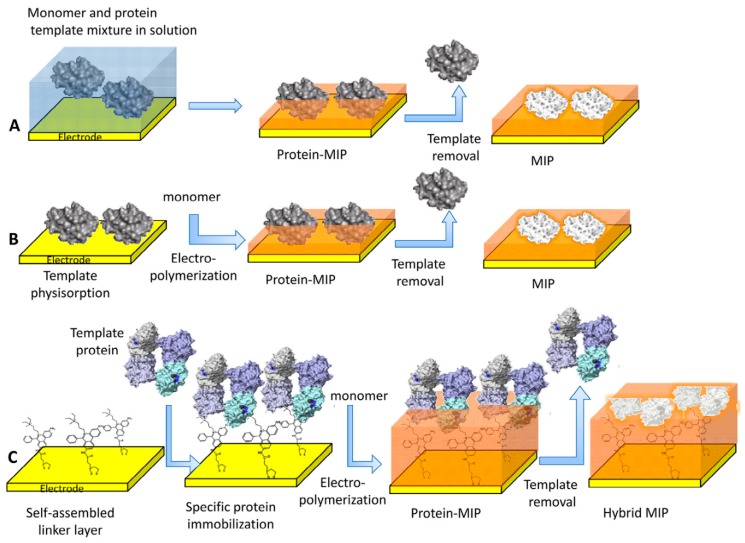
Sketches of various surface imprinting methodologies for the electrosynthesis of MIP films for selective recognition of proteins: electropolymerization of a mixture of protein and monomer in solution (**A**); preconcentration of the protein (peptide in case of epitope imprinting) on the surface of an electrode either by physisorption (**B**) or by using a self-assembled anchor layer for oriented immobilization of the protein (e.g., a weak inhibitor of an enzyme) (**C**). Image reproduced from [110] with permission.

Molecular imprinted polymers have a structure that is characterized by synthetic receptors that are prepared by the molecularly imprinting process [109,110,111]. The polymerization is performed in the presence of the target molecules that act as a shape around which the recognition sites are formed. For sensing applications, MIPs outperform biological receptors in term of durability, low cost and chemically stability. The size of target molecules varies from less than 1 nm for the smallest ones, such as ascorbic acid [112], to about 10 nm for proteins or other high molecular weight biocompounds. If it is difficult to define a recognition site smaller than 1 nm as a nanostructure, certainly an imprint of a macromolecule of 10 nm can be considered a nanostructure. For this reason, this review is focused on MIPs for large molecules or with a nanostructured morphology. Figure 12 shows the main strategies for the electrochemical synthesis of MIPs suitable for protein detection.

Sensors based on MIPs exploit a signal transduction that is mainly electrochemical, though surface plasmon resonance and quartz microbalance have been also reported. Anyhow, a thin film of polymer must be usually deposited on a surface and, for this reason, electrosynthesis offers some intrinsic advantages in addition to the ones previously described in the Section 4.3. Electrochemical polymerization does not need an initiator, and this is a key point when the synthetic receptor must be built around a huge biological molecule, such as a protein [113]. In fact, several initiators are reactive compounds that can modify the ternary structure of biological molecules during polymerization, and, consequently, the resulting recognition sites would take a wrong shape. In addition, the film thickness is a key parameter for the synthesis of some MIPs for protein detection synthesized based on different electrochemical techniques. In addition, the film thickness is a key parameter for the sensor working and the electrochemical polymerization is the best approach to finely control it [114,115], e.g., by varying the number of cycles during a potentiodynamic synthesis. Table 1 reports the synthesis of some MIPs for proteins detection based on different electrochemical techniques. 

The electrochemical polymerization is usually performed in water to preserve the ternary structure of biomolecules and, at the same time, the template removal should be carried out in a way that does not alter the structure of the recognition sites. The formation of a prepolymerization complex can help the synthesis, and it can be obtained by simply dissolving the target molecules in the monomer solution [110,116]. Moreover, different strategies can be followed to increase the number of active sites in the polymer. The analytes can be concentrated on the electrode surface by applying a potential that is the opposite one of the target molecule [117]. However, applying an anodic potential can lead to a depletion of template molecules close to electrode surface. A pulsed potential ramp can overcome this issue. Another possible solution is anchoring the target compound to the electrode surface through a self-assembled layer which leads to a significant enhancement of analytical performance [118].

The current research trend is to increase the surface area of MIP-based sensors because it is strictly linked to binding capabilities. However, the requirement of a thin film is maintained because a high accessibility to the synthetic receptor is mandatory to have a quick sensor response [114]. The electrochemical polymerization can be carried out with a sacrificial scaffold to obtain ordered nanostructures in the form of nanotubes, nanowires or nanoparticles, as above reported. The nanostructured MIPs exhibit higher performance than thin film materials in terms of binding ability. Suriyanarayanan et al. [126] clearly show the advantages of nanostructured MIPs when the transduction is based on quartz microbalance. The sensitivities for a MIP nanowire is about 20 times higher than that of a sensor based on a MIP thin film. Table 2 reports some information about nanostructured MIPs found in the literature.

## 5. Conclusions and Future Perspectives

In this review the main electrochemical techniques employed to synthesize nanomaterials to be used as coatings of conductive surfaces for the development of electrochemical sensors have been described. When the electrodeposition is feasible the adhesion of the coating is better than that achievable with other modification techniques, and the nanostructures are already anchored to a support without the use of chemical or binding agents that could interfere with subsequent sensing applications. However, electrochemical depositions produce a low amount of material on the electrode surface making the characterization more complex. For example, the determination of size distribution for chemically synthesized metal NPs can be easily obtained with dynamic light scattering, a technique that cannot be used for coating investigations. In the same way, the structural and elemental analysis is difficult to accomplish for LDHs unless the electrosynthesis is repeated several times on the same electrode. Moreover, the molecular weight distribution is rarely determined for electrodeposited polymers. The research efforts should be devoted to the progress of characterization tools, in order to better identify the correlation between sensing performance and structural/chemical properties.

The major advantage in the use of nanomaterials, independently of the kinds of chemical modifiers employed, is the big increase of the electrochemically active area and the better accessibility of the analyte to the electrode surface, as demonstrated by the improvement of both sensitivity and limit of detection. The available larger area is also beneficial for the immobilization of biomolecules and, therefore, in the fabrication of biosensors. Moreover, the features of the nanostructure can lead to an increased selectivity of the sensors which is generally the major drawback of the electrochemical ones. As an example, a different morphology and preferential crystalline faces of Au nanomaterials are able to induce selectivity for the detection of chemically similar analytes. In case of LDHs the different standard potential of the redox active metal makes possible the discrimination between oxidizable molecules containing only one or more hydroxyl groups. As to polymeric modifiers, MIPs can be considered the most attractive materials to increase selectivity due to their recognition sites.

As far as metal nanomaterials are concerned the research could be especially focused on multiple metal composites so to improve the stability and the selectivity of the (bio)sensors thanks to the different electrochemical reactivity.

As to LDHs the increase of electrical conductivity and accessible area which can be obtained with the insertion of carbon based nanomaterials should address the research towards new electrochemical procedures for the one-step deposition of the composites.

At present, MIPs are particularly employed as electrode modifiers for the detection of stable macromolecules. In the next future, their use will be probably expanded to the determination of proteins with unstable ternary structure.

All these facts lead to the conclusion that the combination of electrochemistry with nanotechnology will find more and more applications for sensing in the next future. Although the fascinating features of electrochemical syntheses, their large use is hindered for mass production as it is very hard to perform if compared to other methods such as inkjet printing, spin coating or roll-to-roll production. Research efforts should be devoted to scale up the electrochemical syntheses of those devices that offer sensing ability that can be hardly reached with other approaches.

## Figures and Tables

**Table 1:** MIPs for protein detection.

Polymerization	Monomer	Analyte	Detection	Ref.
PT +0.9 V	EDOT	Avidin	Microgravimetric chip	[114]
PD 0–+1.2 V 50 mV·s^−1^	*p*-bis(2,2′-Bithien-5-yl)methyl-alanine-5,5′,5′′-methanetriyltris(2,2′-bithiophene)	Human serum albumin	DPV, EIS	[119]
PD −0.7–+0.6 V 0.1 V·s^−1^	Methylene green	Thrombin	EIS	[120]
PD −0.2 V–+1.2 V 100 mV·s^−1^	Pyrrole	Bovine hemoglobin	DPV	[121]
PD 0–+1.1 V 50 mV·s^−1^	*o*-Phenylenediamine	Troponint	CV, DPV	[122]
PD 0–+1.1 V 50 mV·s^−1^	*o*-Phenylenediamine	Troponint	CV, EIS	[116]
PD −0.2–+1.2 V	Pyrrole	Bovine hemoglobin	DPV, EIS	[123]
PD −0.45–+0.55 V 50 mV·s^−1^	Dopamine	Immunoglobulin G	QCM	[124]
PD 0–+0.9 V	Phenol	Ovarian cancer marker	DPV	[125]

PT = potentiostatic synthesis, PD = potentiodynamic synthesis, DPV = differential pulse voltammetry, CV = cyclic voltammetry, QCM = quartz crystal microbalance, EIS = electrochemical impedance spectroscopy.

**Table 2:** Nanostructured MIPs for sensing.

Polymerization	Monomer	Template Structure	Nanostructure	Analyte	Detection	Ref.
PD (from 0 to +1.4 V vs. Ag/AgCl, 50 mV·s^−1^)	2,2′-Bithiophene-5-carboxylic acid	Porous crystalline Metal−Organic Framework	Molecular cavities	Lipocalin	FET	[127]
PD from 0.0 to +0.8 V	Pyrrole	No template	Nanowires	Dopamine	DPV	[128]
PD from −0.4 to +1.6 V at 50 mV·s^−1^	3-Thienyl-boronic acid (3-TBA) and 3-thiophene acetic acid (3-TAA), and thiophene	Micelle deposition, alumina template	Nanoparticles, nanowires	Aspirin	QCM	[126]
PD from 0 to +1.2 V 100 mV·s^−1^	Pyrrole	Deposition on ZnO nanorods	Nanorods	Epinephrine	DPV	[129]
PD from −0.4 V to +1.0 V (vs. Ag/AgCl, scan rate 50 mV·s^−1^	Aniline	Nanoporous alumina membranes	Nanowire	Catechol	CV	[130]
PD from 0 V to +1.1 V at a scan rate of 100 mV·s^−1^	Terthiophene-based monomer with an acetic acid moiety	Polystyrene microbeads	Nanonetwork	Aspartame	QCM	[131]
PT +1.3 V	2,3′-Bithiophene	Nanoparticles	Nanonetwork	Human serum albumin	EG-FET	[132]
PD from −0.2 to +1.2 V 100 mV·s^−1^	Pyrrole	SiO_2_–CHO microsphere	Nanonetwork	Bovine hemoglobin	DPV	[123]

FET = field effect transistor, EG-FET = extended-gate field effect transistor.
